# MiR‐646 suppresses proliferation and metastasis of non‐small cell lung cancer by repressing FGF2 and CCND2

**DOI:** 10.1002/cam4.3062

**Published:** 2020-04-29

**Authors:** Jing Wang, Huizhen Shu, Shuigen Guo

**Affiliations:** ^1^ Department of Respiratory Disease Jinshan Hospital of Fudan University Shanghai China; ^2^ Xuanqiao Community Health Service Center Shanghai China; ^3^ Department of Respiratory Disease Shanghai Pudong Hospital Fudan University Pudong Medical Center Shanghai China

**Keywords:** CCND2, FGF2, miR‐646, non‐small cell lung cancer

## Abstract

MicroRNA‐646 (miR‐646) has been implicated in several other cancers; however, its functional mechanism in non‐small cell lung cancer (NSCLC) remains unclear. In this study, we observed the downregulation of miR‐646 expression in NSCLC tissues and cell lines. Low‐level expression of miR‐646 was associated with metastasis and stage of NSCLCs. Functional assays showed that overexpression of miR‐646 could suppress NSCLC cell proliferation, clonogenicity, invasion, and inhibit epithelial‐mesenchymal transition (EMT), whereas decreased miR‐646 expression showed the opposite effects. Importantly, miR‐646 overexpression attenuated in vivo tumor growth and metastasis in nude mice models. Mechanically, miR‐646 directly targeted and suppressed fibroblast growth factor 2 (FGF2) and cyclin D2 (CCND2) expression. Reintroduction of FGF2 and CCND2 attenuated miR‐646‐mediated suppression of proliferation and invasion in NSCLC. Collectively, these results demonstrate that miR‐646 acts as a tumor suppressor in NSCLC by targeting FGF2 and CCND2, and may serve as a therapeutic target for patients with NSCLC.

## Graphical abstract

MiR‐646 is downregulated in non‐small cell lung cancer (NSCLC) and could suppress the proliferation and EMT‐induced metastasis of NSCLC by suppressing FGF2 and CCND2. Thus, downregulation of miR‐646 can play a central role in tumor progression and may be a potential therapeutic target for NSCLC.

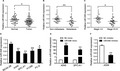

## 1 INTRODUCTION

Lung cancer, a common invasive malignancy, has become the leading cause of cancer‐related death worldwide.^1^ Based on pathological and etiological characteristics, lung cancer can be grouped into two major types, that is, small cell lung cancer (SCLC) and non‐small cell lung cancer (NSCLC).^2^ The latter includes adenocarcinoma, squamous cell carcinoma, and large cell carcinoma, and accounts for 85%‐95% of lung cancers.^3^ The dominating treatment for NSCLC is surgical resection combined with both chemo‐ and radiation therapy to date. However, owing to the high rates of metastasis and recurrence, the prognosis of NSCLC remains poor.^4^ Besides, patients with NSCLC are commonly diagnosed at a later stage. Hence, there is a pressing need for the exploration of new biomarkers for the precise diagnosis and novel treatment strategies of NSCLC.

MicroRNAs (miRNAs) are a group of small, single‐stranded, noncoding RNAs, which consist of 19‐25 nucleotides (~22 nt^5^). They are considered as one of the pivotal post‐transcriptional regulators of gene expression by binding to the 3’‐untranslated region (UTR) of target mRNAs to modulate mRNAs translation.^6^ It has been testified that numerous microRNAs play essential roles in tumorigenesis, and believed to act as tumor suppressors or oncogenes in a variety of other human cancers.^7^, ^8^, ^9^ MiR‐646 is downregulated and correlated with malignant progression in specific carcinoma, including clear cell renal carcinoma, gastric, and endometrial cancers.^10^, ^11^, ^12^ Also, it has been shown to contribute to tumorigenesis of pancreatic cancer.^13^ The real reason for the dual effects of miR‐646 is not apparent yet, which in part might be due to organ‐specific actions and the different cellular contexts of tumors.

Here, we investigated the potential role and underlying mechanisms of miR‐646 in the development of NSCLC. We found that miR‐646 was downregulated in tissues from NSCLC patients and cell lines. Besides, both the gain‐ and loss‐of‐function assays disclosed that miR‐646 suppressed the proliferation and invasion of NSCLC cells in vitro and in vivo by downregulating fibroblast growth factor 2 (FGF2) and cyclin D2 (CCND2). These results provide new insight into the pathophysiology of NSCLC.

## 2 MATERIALS AND METHODS

### 2.1 Patients and specimens

Primary tumor tissues and their corresponding adjacent nontumorous lung specimens were obtained from 49 patients who suffered from NSCLC and underwent lung resection at Jinshan Hospital of Fudan University. Before the surgery, none of the patients had received any cancer treatments like radiotherapy or chemotherapy. Fresh specimens were immediately frozen in liquid nitrogen, and then transferred to −80°C for subsequent experiments. The research protocol was approved by the Institutional Ethics Committee of Jinshan Hospital of Fudan University, and human tumor tissues were obtained with informed consent.

### 2.2 Cell lines and culture conditions

Four NSCLC cell lines (A549, SPC‐A1, H1299, and PC‐9) and a normal human bronchial epithelial cell line (BEAS‐2B) were purchased from Cell Bank of Type Culture Collection of the Chinese Academy of Sciences (Shanghai, China). All cells were cultured in RPMI 1640 medium (Gibco) with 10% fetal bovine serum (FBS, Gibco), penicillin (100 U/mL), and streptomycin (100 µg/mL). Cells were preserved at 37°C in a 5% CO_2_ humidified atmosphere.

### 2.3 RNA extraction and quantitative real‐time PCR (qRT‐PCR) and Western blotting analysis

Total RNA was extracted from cell lines and frozen tumor specimens using Trizol reagent (Life Technologies). The qRT‐PCR assays were carried out to detect the expression of miR‐646, FGF2, and CCND2 using the PrimeScript RT reagent Kit and SYBR Premix Ex Taq (GeneCopoeia) according to the manufacturer's instructions. MiRNA and target mRNA expression levels were normalized against the endogenous U6 small nuclear RNA (U6 snRNA) and GAPDH, respectively. The data were analyzed using the 2^−ΔΔCT^ method. For Western blotting analysis, cellular proteins were extracted from the cultured cells with a modified RIPA buffer with 0.5% sodium dodecyl sulfate (SDS) and the proteinase inhibitor cocktail (Complete Mini, Roche). Protein samples were separated in SDS‐PAGE gel and transferred to a PVDF membrane (Immobilon P‐SQ, Millipore). After blocked in PBS/Tween‐20 with 5% nonfat milk at room temperature, the membranes were incubated with FGF2, CCND2, E‐cadherin, and vimentin antibodies (Abcam). Protein visualization was carried out using ECL reagents (Pierce).

### 2.4 Oligonucleotide transfection

The miR‐646 mimic, inhibitor, and their negative control oligonucleotides were purchased from RiboBio (Guangzhou). The mutated miR‐646 mimic (miR‐646‐mut) was also obtained from RiboBio. The siRNAs targeting FGF2 and CCND2 were synthesized by HanBio. NSCLC cell lines (A549 and SPC‐A1) were transfected with miR‐646 mimic, miR‐646 inhibitor, or their respective control using Lipofectamine 2000 transfection reagent. After 48 hours transfection, the cells were used for further experiments.

### 2.5 Cell proliferation and colony formation assays

Cell proliferation was monitored using a CCK‐8 test. Cells were seeded into 96‐well plates at 5 × 10^3^ per well with 100 μL RPMI 1640 medium. After cultured for overnight, cells were transfected with miR‐646 mimic or inhibitor and then recultured for another 24, 48, 72, and 96 hours. Then, 10 μL of CCK‐8 solutions (Beyotime) was added into each well for cell staining and incubated for 4 hours at 37°C. Finally, the OD value was measured at 490 nm with a Synergy plate reader (Biotech). For colony formation assay, approximately 400 transfected cells for each well were seeded in a 6‐well plate and incubated for 12 days. Afterward, PBS and methanol were used to wash and fix the cells, respectively, and finally, cells were visualized by staining with 0.5% crystal violet. The visible colonies were counted with an ELIspot Bioreader 5000.

### 2.6 BrdU incorporation and immunostaining

This assay was performed according to a standard method, as described previously.^14^ Briefly, cells were seeded in the 6‐well plate at a density of 1 × 10^4^ cells/well on sterilized coverslips. After 48 hours, BrdU (Sigma‐Aldrich) was added to the medium at a final concentration of 10 μmol/L and incubated at 37°C for 5 hours. Cells were fixed in cold 70% ethanol for 5 minutes. Immunofluorescence was performed using a mouse anti‐BrdU antibody (BD Biosciences) to visualize the incorporated BrdU, followed by 45 minutes incubation with secondary antibody. VECTASHIELD mounting medium with DAPI was used to stain the nuclei.

### 2.7 Transwell invasion assay

Invasion of NSCLC cells with indicated transfection was performed by Transwell chambers (Corning Costar Corp, Cambridge, MA) with Matrigel (BD Biosciences) and 8‐µm porous membranes as previously described.^15^ Cells (5 × 10^4^) were plated into the top side of the chamber in 100 μL of RPMI 1640 medium. The lower chamber was inoculated with 400 μL RPMI 1640 medium, supplemented with 10% FBS in 24‐well plate. After incubation for 24 hours, the uninvaded cells inside the upper chamber were erased with cotton swabs. The invaded cells on the bottom side of the membrane were fixed and stained with 0.1% crystal violet, photographed, and then counted in five fields randomly.

### 2.8 Dual‐luciferase reporter assay

Fragments of *FGF2* and *CCND2* 3′UTR sequences containing the putative binding site for miR‐646 were cloned into the psiCHECK‐2 luciferase reporter vector. A549 and SPC‐A1 cells were planted into 24‐well plate and cultured for 24 hours. MiR‐646 mimic or miR‐646‐mut mimic were co‐transfected with appropriate reporter plasmids into A549 and SPC‐A1 cells, respectively, using the Lipofectamine 2000 reagent. After 48 hours, cells were washed with cold PBS and lysed with luciferase assay buffer. Subsequently, the luciferase activity was measured using the Dual‐Luciferase Reporter Assay Kit (Promega) by following the manufacturer's protocol.

### 2.9 Lentivirus production

The pre‐miR‐646 sequence was synthesized and cloned into the pCDH lentiviral vector (System Biosciences, California, USA) to generate pCDH‐miR‐646. The validated vector and the lentivirus packaging vectors (pMD and psPAX2) were co‐transfected into 293T cells using Lipofectamine 2000 reagent. After 48 hours, virus particles were harvested and then purified. A549 cells were infected with recombinant lentivirus‐transducing units plus 8 mg/mL Polybrene (Sigma‐Aldrich).

### 2.10 In vivo tumor growth and metastasis assays

All animal experiments were approved by the Animal Care and Use Committee of Fudan University. BALB/c nude mice (aged 4‐5 weeks) were upraised in barrier facilities on a 12 hours light/dark cycle. A549 cells infected with miR‐646 or control lentivirus (5 × 10^6^ in 100 μL sterile PBS) were subcutaneously injected into the flanks of female BALB/c nude mice (n = 5). Tumor size was detected every week by measuring the length and width with calipers, and the volumes were evaluated with the formula: tumor volume = (width^2^ × length)/2. Four weeks later, mice were killed, and the tumor sections were gathered for the experiments. For in vivo metastasis assay, a total of 5 × 10^6^ cells were injected into the caudal vein of nude mice. After 40 days, the mice were killed and lung tissues were dissected for histological examination.

### 2.11 Immunohistochemistry analysis

Tumor sections (thickness, 5 μm) taken from nude mice were fixed in 4% formalin and then embedded in paraffin, The sections were then incubated with E‐cadherin and vimentin primary antibodies for 12 hours at 4°C after blocking. Followed by washing with PBS, sections were incubated with HRP‐polymer‐conjugated secondary antibody at 37°C for 30 minutes and washed with PBS for three times. Then, sections were immersed in a 3, 3‐diaminobenzidine solution for 3 minutes and the nuclei were counterstained with 10% Mayer hematoxylin. Finally, the parts were dehydrated and mounted in crystal mount.

### 2.12 Statistical analysis

All data shown are presented as the mean ± SEM of three independent experiments. The Student *t* test was performed for comparisons between treated groups relative to their paired controls. Pearson's correlation analysis was used to determine the relationship between miR‐646 expression and the expression of FGF2 and CCND2 in NSCLC tissues. Statistical significance was assumed for *P* < .05.

## 3 RESULTS

### 3.1 MiR‐646 expression is downregulated in NSCLC tissues and cell lines

**FIGURE 1:**
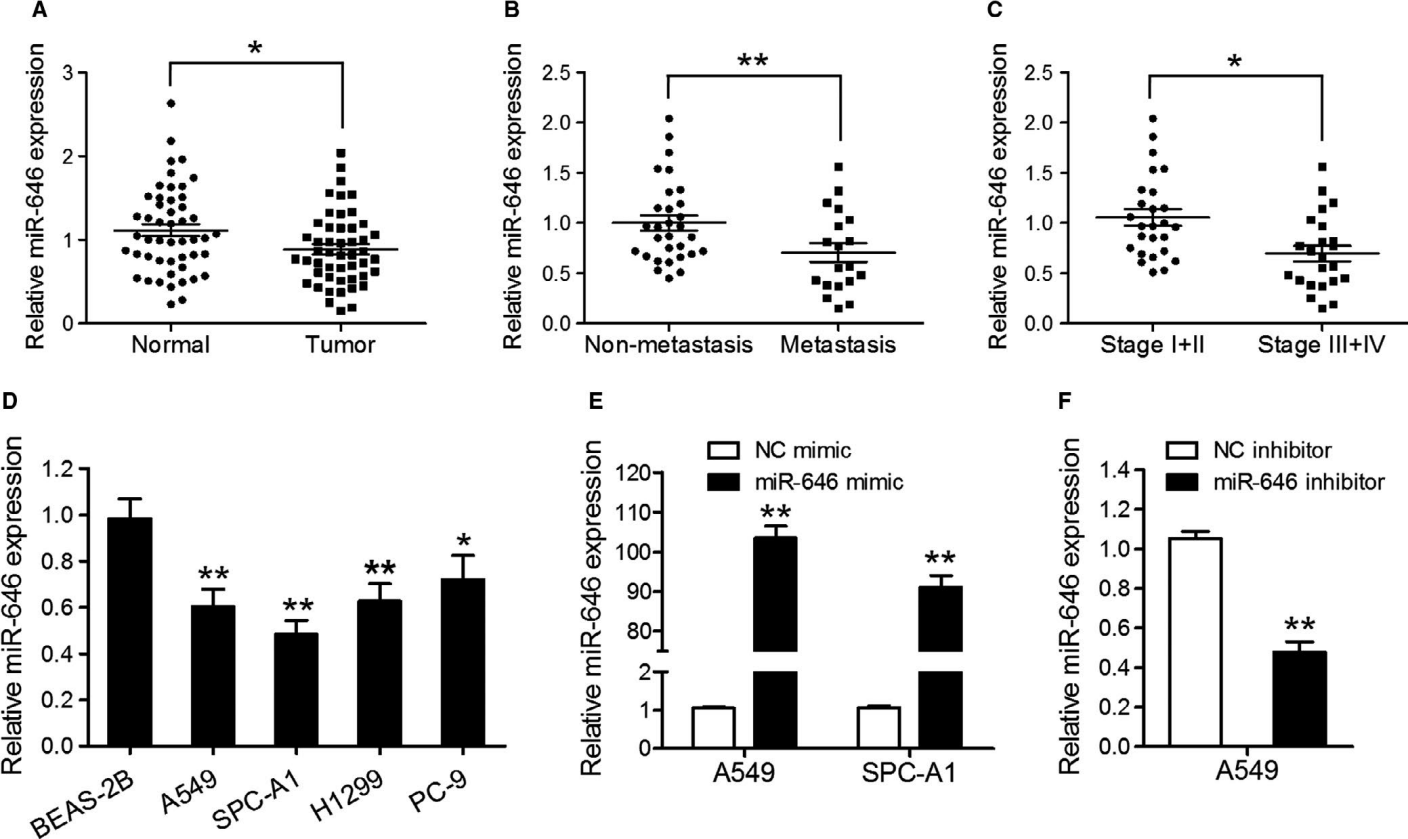
MiR‐646 is downregulated in NSCLC tissues and cell lines. (A) qRT‐PCR assay was performed to quantify miR‐646 expression in 49 pairs of NSCLC tissues and adjacent noncancerous lung tissues. (B) miR‐646 expression in metastatic and nonmetastatic NSCLCs. (C) miR‐646 expression in different clinical stages of NSCLCs. (D) The endogenous levels of miR‐646 in four NSCLC cell lines (A549, SPC‐A1, H1299, and PC‐9) and the normal human bronchial epithelial cell line (BEAS‐2B). (E) qRT‐PCR analysis of miR‐646 expression in A549 and SPC‐A1 cells transfected with miR‐646 mimic or NC mimic. (F) qRT‐PCR analysis of miR‐646 expression in A549 cells transfected with miR‐646 inhibitor or NC inhibitor. *P < .05, **P < .01

Previously, miR‐646 has been shown to underexpressed in lung cancer tissues.^16^ To confirm this, we evaluated the expression levels of miR‐646 in 49 pairs of human NSCLC tissues and adjacent noncancerous lung tissues by qRT‐PCR. As shown in Figure 1A, the mean expression level of miR‐646 was lower in NSCLC tissues compared to matched noncancer tissues. Correlation analysis showed that the expression of miR‐646 in NSCLCs was associated with metastasis and TNM stage (Table 1). Likewise, miR‐646 expression was lower in NSCLCs which displayed the lymph node metastasis than their counterparts (Figure 1B) and decreased statistically with increasing stage of NSCLCs (Figure 1C). Furthermore, we compared a normal human bronchial epithelial cell line (BEAS‐2B) with a panel of NSCLC cell lines (A549, SPC‐A1, H1299, PC‐9) for the expression of miR‐646 and found generally decreased miR‐646 expression in the NSCLC cell lines (Figure 1D). These results affirmed that miR‐646 expression is universally downregulated in NSCLC.

**TABLE 1:** Correlation between miR‐646 expression and clinicopathological features of NSCLCs

Characteristics	Case	miR‐646 expression (%)		*P* value
		Low	High	
Sex				
Male	29	15	14	1.000
Female	20	10	10	
Age (year)				
<60	22	11	11	1.000
≥60	27	14	13	
Tumor size				
<5 cm	28	13	15	.567
≥5 cm	21	12	9	
Histological type				
Adenocarcinoma	29	15	9	.156
Squamous cell carcinoma	20	10	15	
Lymph node metastasis				
Negative	33	13	20	
Positive	16	12	4	.032
TNM stage				
I + II	31	12	19	
III + IV	18	13	5	.038

### 3.2 MiR‐646 inhibits the malignant phenotype of NSCLC in vitro

**FIGURE 2:**
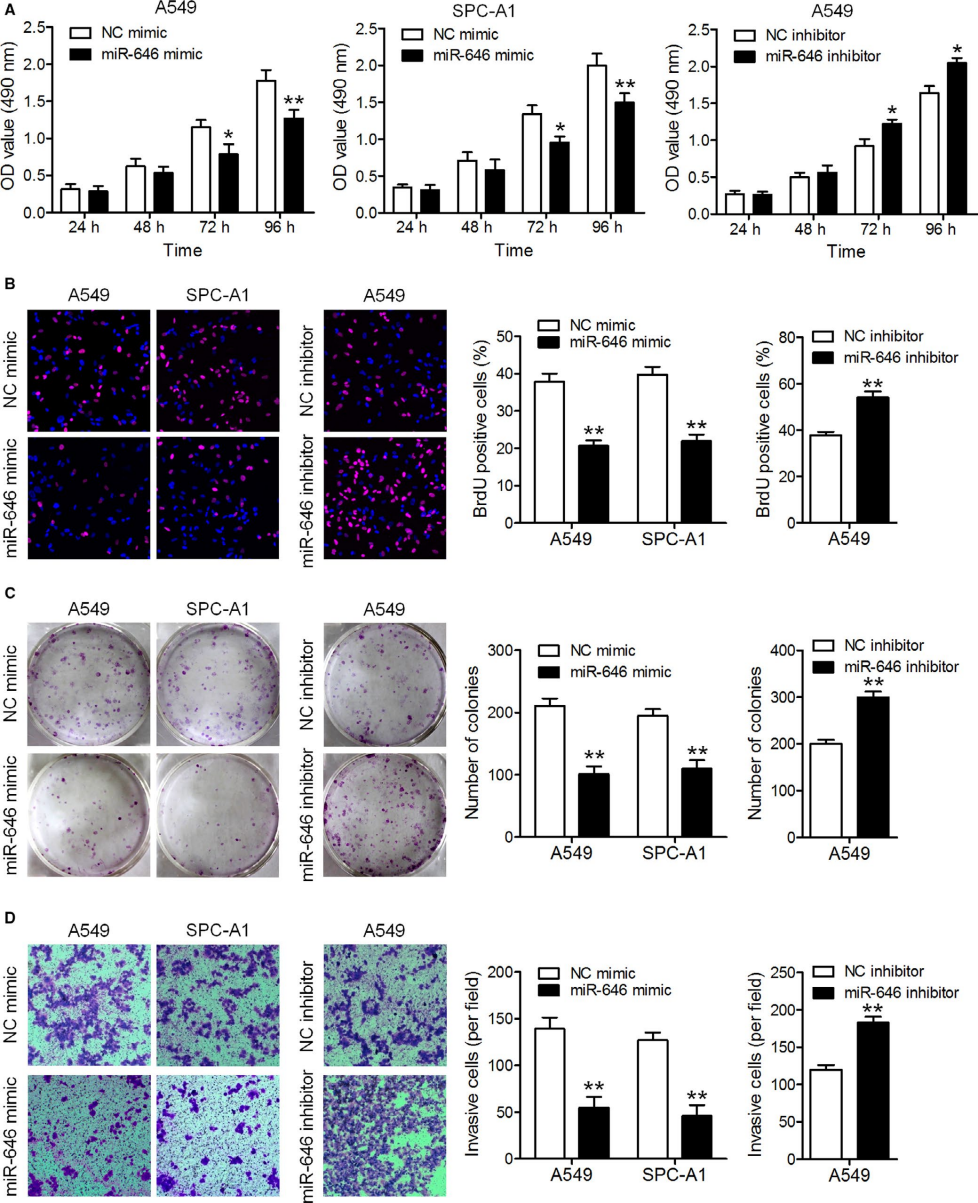
MiR‐646 suppresses the proliferation and invasion of NSCLC cells in vitro. (A) A549 and SPC‐A1 cells were transfected with miR‐646 mimic or its inhibitor, and cell proliferation was evaluated by CCK‐8 assay. (B) Representative micrographs (left) and quantification (right) of BrdU‐incorporating cells transfected with miR‐646 mimic or its inhibitor. (C) Colony formation assay. Representative images (left) and quantification (right) of colony formation of the transfected cells. (D) Representative images (left) and quantitation (right) of the Transwell assay. *P < .05, **P < .01

To evaluate the role of miR‐646 in NSCLC, we investigated the effects of miR‐646 on the proliferation and invasion of NSCLC cells. A549 and SPC‐A1 cells were selected for the transfection of miR‐646 mimic or NC mimic. The transfection efficiency of the cell lines was validated using qRT‐PCR (Figure 1E). CCK‐8 assay showed that overexpression of miR‐646 suppressed the growth of A549 and SPC‐A1 cells compared to the negative control (Figure 2A). Following miR‐646 overexpression in A549 and SPC‐A1 cells, consistently, BrdU incorporation was drastically reduced. Also, miR‐646 overexpression was found to decrease colony formation in both A549 and SPC‐A1 cells (Figure 2B). These results indicate that miR‐646 can enhance the growth and clonogenic potential in A549 and SPC‐A1 cells. Furthermore, a Transwell invasion assay was performed to evaluate the roles of miR‐646 in NSCLC cell invasion. The result of Figure 2C showed that overexpression of miR‐646 remarkably restrained the invasion of A549 and SPC‐A1 cells. In contrast, miR‐646 knockdown using miR‐646 inhibitor in A549 cells significantly increased cell proliferation besides invasion as compared to the negative control (Figure 2A‐D). Overall, these data show that miR‐646 could regulate several malignancy parameters in human NSCLC cells.

### 3.3 MiR‐646 suppresses tumor growth and metastasis of NSCLC cells in vivo

**FIGURE 3:**
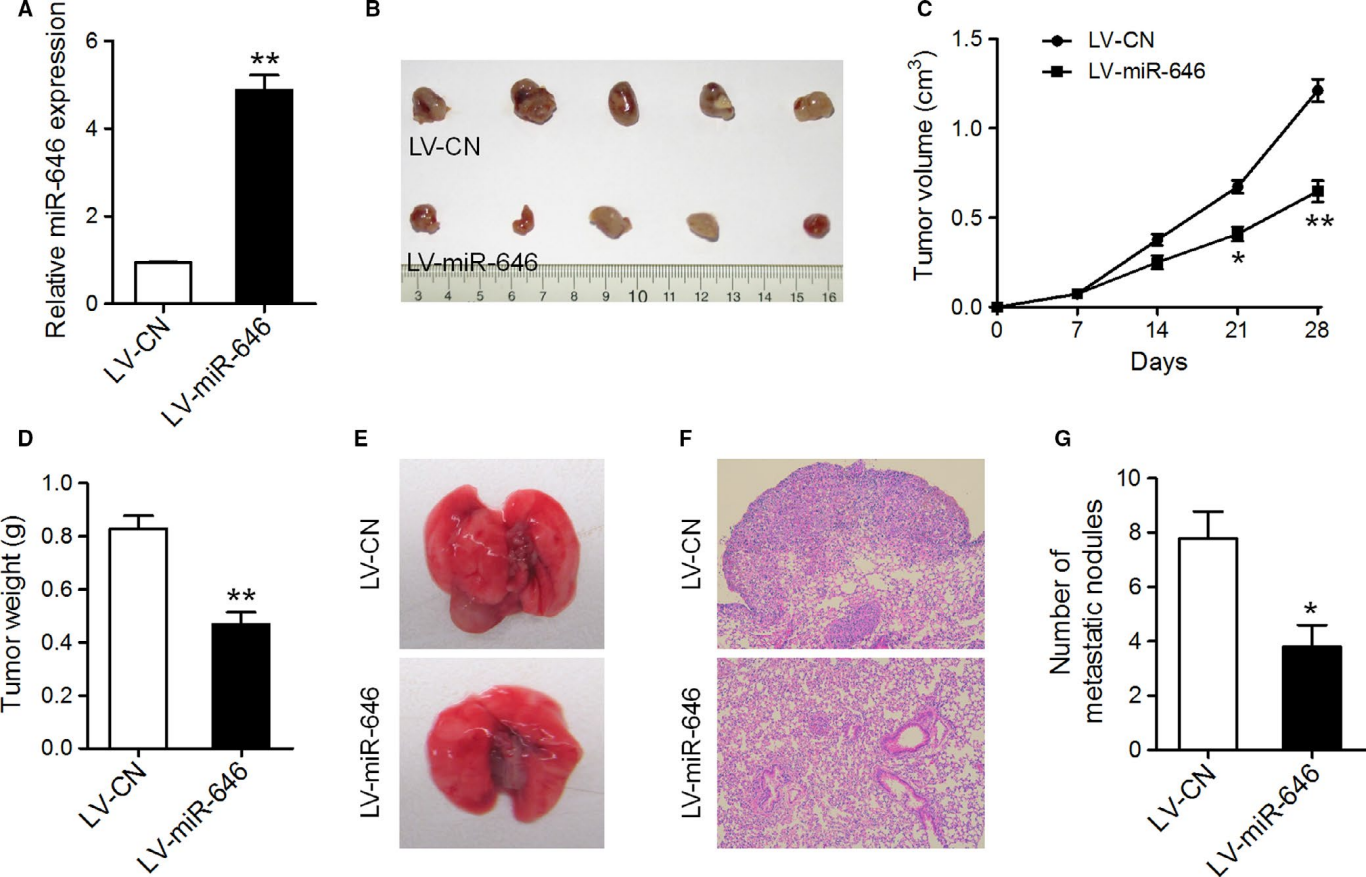
MiR‐646 attenuated tumor growth and metastasis in vivo. (A) qRT‐PCR analysis of miR‐646 expression in A549 cells infected with miR‐646 overexpression or control lentivirus. (B) Flank tumors were established in nude mice after injection with miR‐646 overexpression or control cells. The mice were killed 4 weeks after flank injection and representative tumors were shown. (C) Growth curves of xenograft tumors. (D) Tumor weight. (E) Representative images of lungs isolated from mice that received tail vein injection of miR‐646 overexpression or control cells. (F) Representative images of HE staining of lungs. (G) The numbers of pulmonary metastatic nodules in the lungs. *P < .05, **P < .01

Next, we examined whether miR‐646 could affect tumorigenicity and metastasis in vivo. A549 cells overexpressing miR‐646 through lentivirus infection and the control cells were injected subcutaneously into the nude mice (Figure 3A), and these mice were monitored closely for tumor growth for 4 weeks. The results illustrated that tumors derived from miR‐646 overexpression cells were significantly smaller than those derived from control cells, both in terms of tumor volumes and weights (Figure 3B‐D). To further evaluate the effect of miR‐646 on NSCLC metastasis in vivo, miR‐646 overexpression and control cells were injected into nude mice through the tail vein. We found that A549 with miR‐646 overexpression yielded less pulmonary nodules than the control cells (Figure 3E‐G). These findings suggest that miR‐646 suppresses tumor growth and metastasis in NSCLC in vivo.

### 3.4 MiR‐646 suppresses EMT in NSCLC cells

**FIGURE 4:**
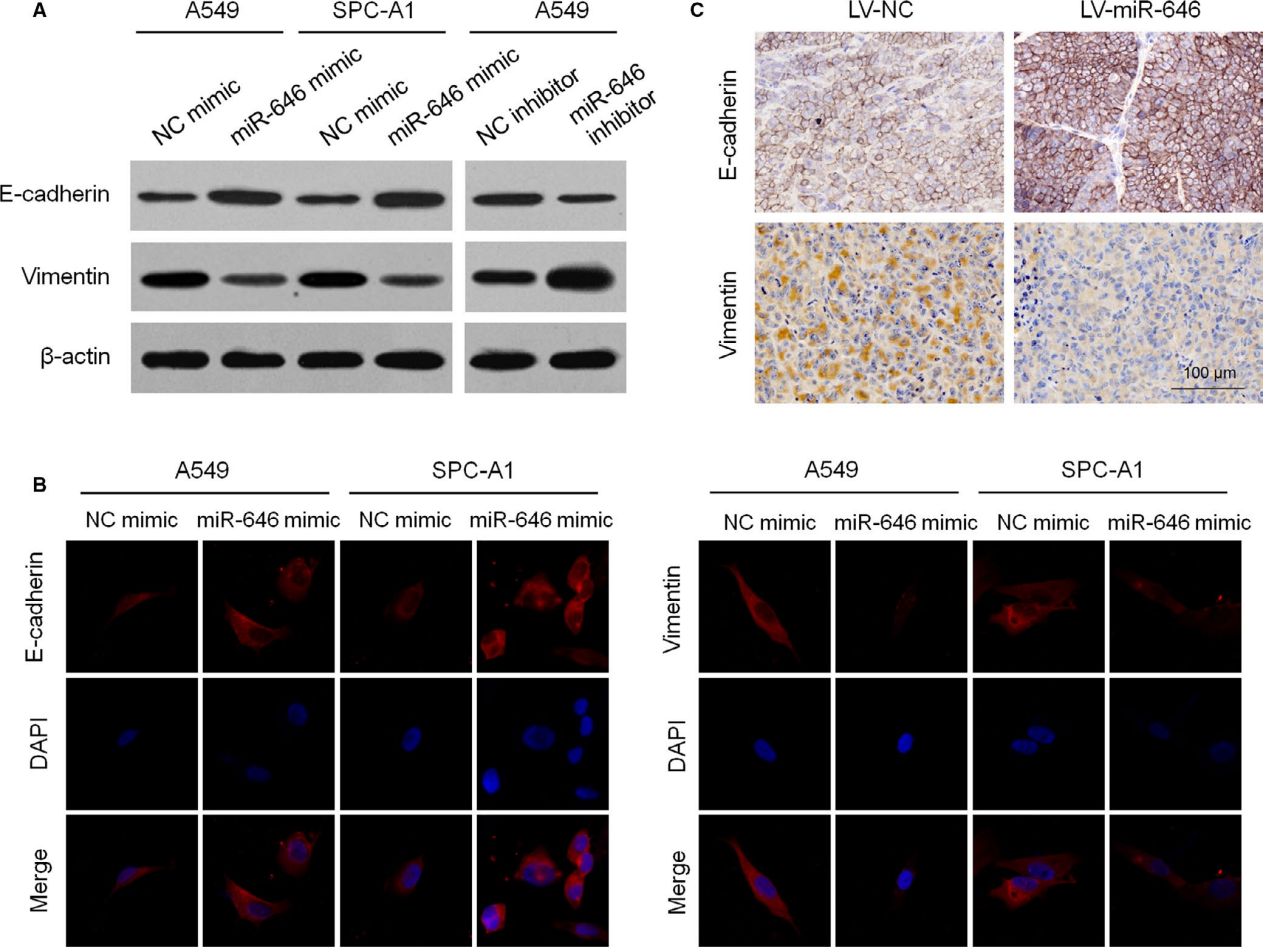
MiR‐646 suppresses EMT process in NSCLC. (A) Western blotting analysis of E‐cadherin and vimentin expression in A549 and SPC‐A1 cells with miR‐646 mimic or inhibitor transfection. (B) Immunofluorescence staining of E‐cadherin and vimentin in A549 and SPC‐A1 cells with miR‐646 mimic transfection. (C) Immunohistochemical staining for E‐cadherin and vimentin in subcutaneous tumors formed with A549 carrying LV‐miR‐646 or LV‐NC. *P < .05, **P < .01

Recent research showed that miR‐646 attenuates EMT process of gastric cancer cells.^11^ To determine whether this phenomenon also exists in NSCLC cells, we examined the expression levels of the epithelial marker E‐cadherin, as well as mesenchymal marker vimentin. We found that E‐cadherin expression was dramatically increased while vimentin expression was downregulated in A549 and SPC‐A1 cells with miR‐646 mimic transfection. However, miR‐646 inhibitor suppressed E‐cadherin expression and induced vimentin in A549 cells (Figure 4A). Consistently, immunofluorescent staining also showed that miR‐646 overexpression leads to the upregulation of E‐cadherin and the downregulation of vimentin (Figure 4B). Furthermore, subcutaneous tumors (originated from A549 cells with miR‐646 overexpression) displayed stronger E‐cadherin and weaker vimentin staining than the control tumors (Figure 4C). These results suggest that EMT process was inactivated with increased miR‐646 expression in NSCLC.

### 3.5 MiR‐646 directly targets the 3'‐UTR of FGF2 and CCND2 to suppress NSCLC cell proliferation and invasion

**FIGURE 5:**
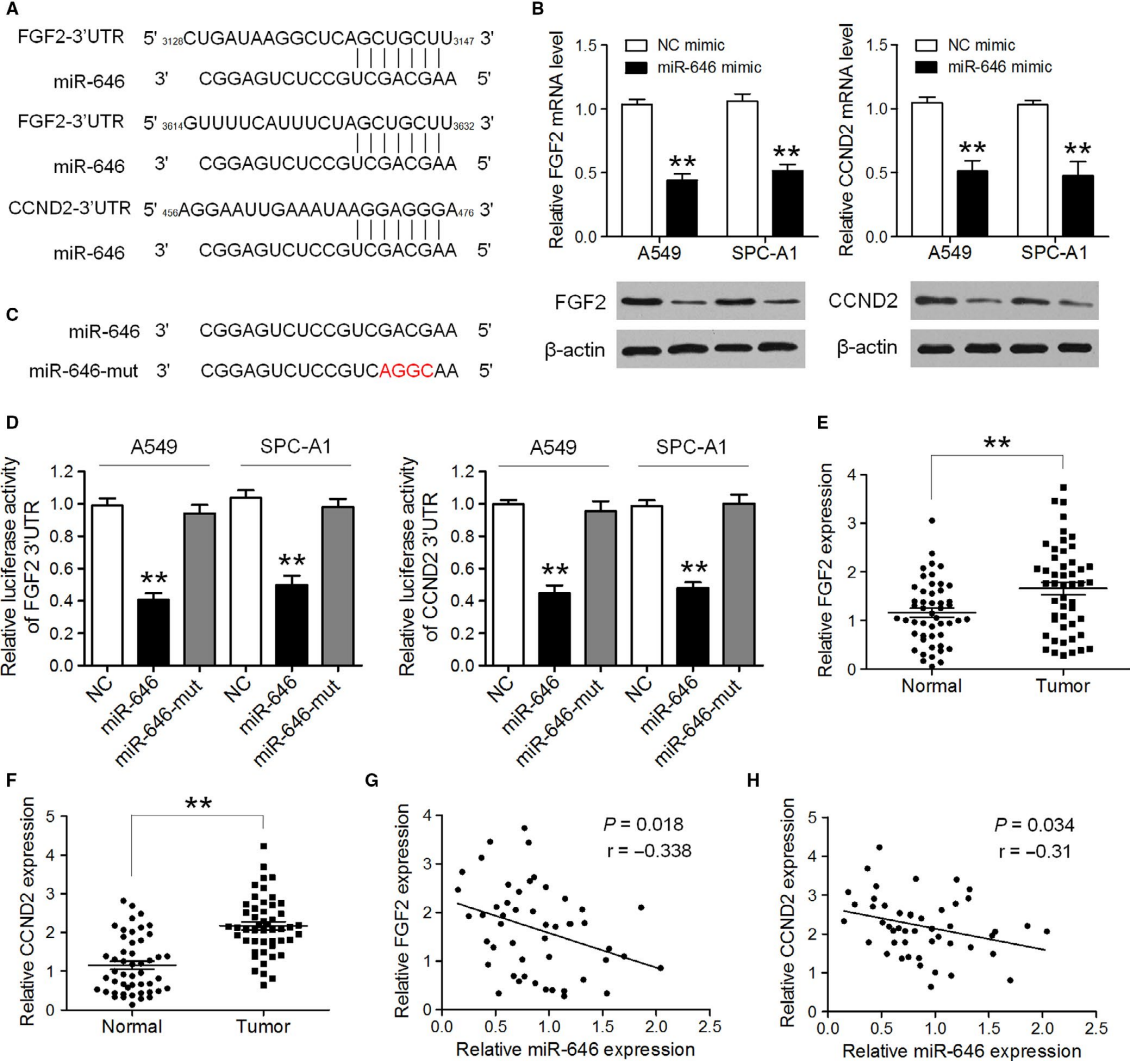
FGF2 and CCND2 were direct targets through which miR‐646 regulated proliferation and invasion of NSCLC cells. (A) Predicted binding sites of miR‐646 in the 3′UTR of *FGF2* and *CCND2*. (B) The expression levels of FGF2 and CCND2 mRNA and protein were detected by qRT‐PCR and Western blotting in A549 and SPC‐A1 cells after transfection of miR‐646 mimic or NC mimic. (C) Sequence of miR‐646‐mut. (D) Luciferase activity was analyzed in A549 and SPC‐A1 cells co‐transfected with miR‐646 mimic or miR‐646‐mut mimic with FGF2‐3'UTR or CCND2‐3'UTR reporters. (E,F)The average expression level of FGF2 and CCND2 in NSCLC tissues and adjacent noncancerous lung tissues. (G,H) Pearson's correlation analyses between miR‐646 expression and the expression of FGF2 and CCND2 in NSCLC tissues. *P < .05, **P < .01

To identify the potential targets of miR‐646 that involved in miR‐646‐mediated regulation of proliferation and invasion of NSCLC cells, we sought publicly available databases (TargetScan, miRDB, and miRanda). *FGF2* and *CCND2* were identified to have the highest potential to bind to miR‐646 (Figure 5A). To confirm whether *FGF2* and *CCND2* were the direct targets of miR‐646, miR‐646 mimic was transfected into A549 and SPC‐A1 cells and was found to markedly downregulate the mRNA and protein levels of FGF2 and CCND2, respectively (Figure 5B). Furthermore, luciferase reporter assays showed that the activity of luciferase linked with the 3′‐UTR of *FGF2* and *CCND2* was repressed in miR‐646 mimic‐transfected A549 and SPC‐A1 cells, compared with those in control cells (Figure 5D). However, mutations brought into the seed sequence of miR‐646 abolished its suppressive effects (Figure 5C,D). We further measured the mRNA levels of FGF2 and CCND2 in NSCLC tissues and adjacent noncancerous lung tissues. The results showed that the average expression level of FGF2 and CCND2 was significantly higher in NSCLC tissues than in matched noncancer tissues (Figure 5E,F). Besides, when FGF2 and CCND2 mRNA levels were plotted against miR‐646 expression, a significant inverse correlation was observed (Figure 5G,H). These results suggest that miR‐646 directly suppresses FGF2 and CCND2 expression in NSCLC.

### 3.6 Suppression of FGF2 and CCND2 is key to the tumor‐repressive function of miR‐646

**FIGURE 6:**
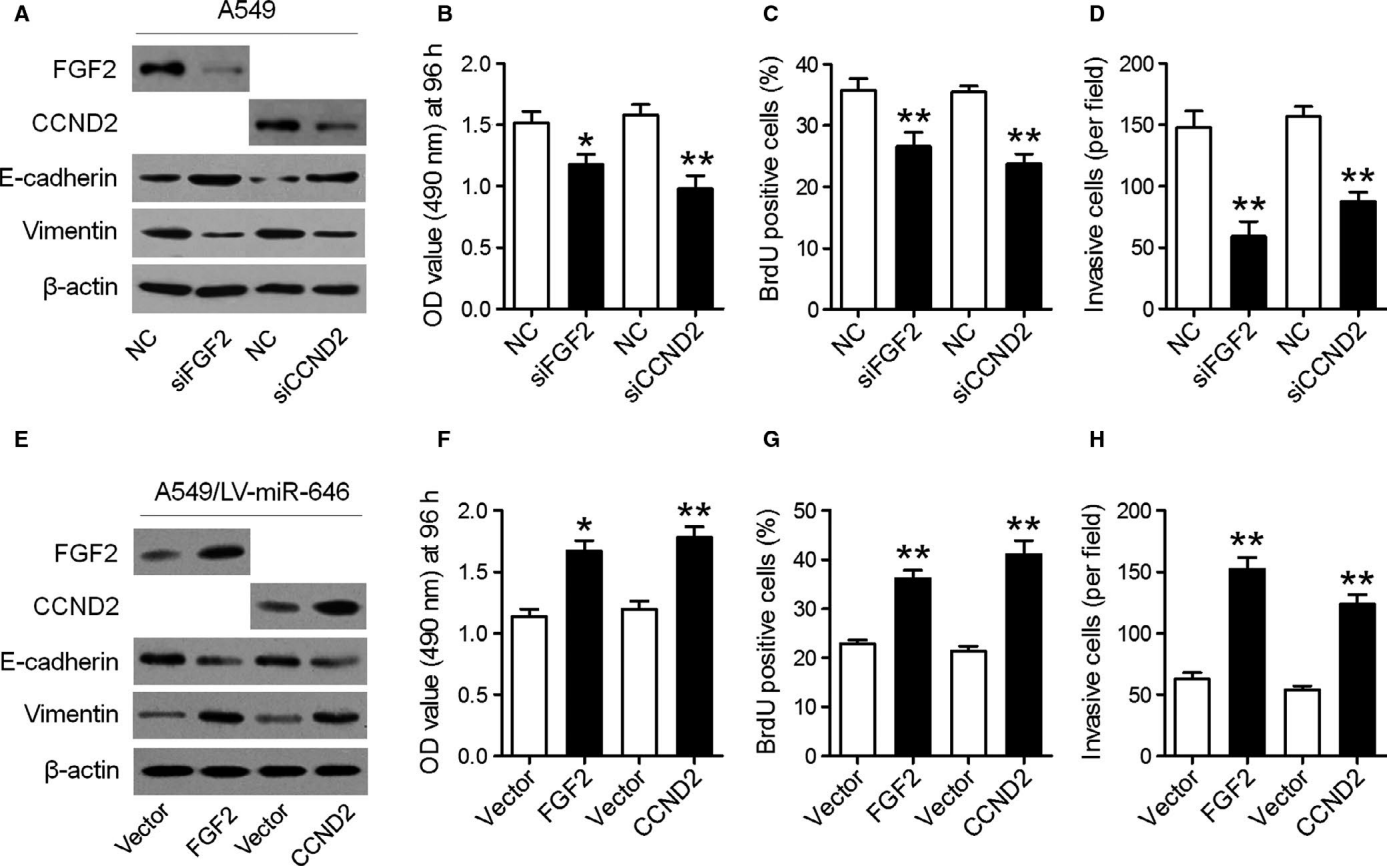
Suppression of FGF2 and CCND2 is key to the tumor‐repressive function of miR‐646. (A) Western blotting analyses of FGF2, CCND2, E‐cadherin, and vimentin in A549 cells transfected with FGF2 siRNA (siFGF2) or CCND2 siRNA (siCCND2). (B) CCK‐8 was used to determine the cell proliferation in different groups. (C) Quantification of BrdU‐incorporating cells. (D) Quantification of invading cells by Transwell assay. (E) Western blotting analyses of FGF2, CCND2, E‐cadherin, and vimentin in A549/LV‐miR‐646 cells after overexpression of FGF2 or CCND2. CCK‐8 (F), BrdU incorporation (G) and Transwell invasion (H) assays were performed. *P < .05, **P < .01

Subsequently, we investigated whether downregulation of FGF2 and CCND2 represses cell proliferation and invasion in NSCLC cells. To this end, siRNAs specifically against FGF2 and CCND2 were transfected in A549 cells, and their effects on cell proliferation and invasion were evaluated (Figure 6A). The results showed that silencing FGF2 and CCND2 expression reduced cell proliferation and invasion, mimicking the biological effects of miR‐646 overexpression (Figure 6B‐D). To further verify that the repression of FGF2 and CCND2 mediated the effect of miR‐646 on the proliferation and invasion of NSCLC cells, we respectively overexpressed FGF2 and CCND2 in A549/LV‐miR‐646 cells (Figure 6E). The results elucidated that the suppressive effects of miR‐646 on cell proliferation and invasion were reversed by transfection of both expression constructs (Figure 6F‐H), suggesting that FGF2 and CCND2 were functionally important for the suppression of miR‐646 on NSCLC cell proliferation and invasion.

## 4 DISCUSSION

NSCLC is the most prevalent lung cancer with metastasis and the leading cause of cancer‐related deaths.^17^ Research and development on new biomarkers and novel therapeutic targets are critically in need owing to the enormous challenge on detection and treatment of the NSCLC. Here, we demonstrated that miR‐646 suppresses proliferation and invasion in NSCLC by directly downregulating the expression of FGF2 and CCND2.

MiR‐646 has been notorious as a tumor suppressor and found to be downregulated in certain malignancies. In clear cell renal carcinoma, downregulated miR‐646 was associated with tumor metastasis through MAPK pathway by targeting NOB1.^10^ MiR‐646 also inhibited cell proliferation and EMT‐induced metastasis by targeting FOXK1 in gastric cancer.^11^ It could interact with HOTAIR and regulate NPM1, thereby governing the viability, migration, and invasion of endometrial cancer cells.^12^ In contrast, miR‐646 was reported as an oncogenic miRNA in pancreatic cancer,^13^ or a critical negative regulator of the EGFR pathway in lung cancer.^16^ However, the potential mechanisms by which miR‐646 affects the malignant phenotype of NSCLC cells remain largely unknown. In this study, we firstly showed that the expression of miR‐646 in NSCLC tissues and cell lines were memorably downregulated. Besides, low‐level expression of miR‐646 is associated with metastasis and stage of NSCLCs. Secondly, we investigated the biological function of miR‐646 in vitro and in vivo to further understand its precise role in NSCLC. Results indicated that miR‐646 could damage the capacity of cell proliferation, colony formation, cellular invasion, suppress EMT of NSCLC cells, and restrain the tumor growth and metastasis in nude mice. These results indicated that miR‐646 played a tumor suppressive role in NSCLC.

To clarify the mechanisms underlying the effects of miR‐646 on NSCLC cell proliferation and invasion, we predicted putative targets of miR‐646 by bioinformatic analysis, and it revealed that miR‐646 could bind to the 3'‐UTRs of FGF2 and CCND2. FGF2 is a member of the FGF family, which is frequently increased in many malignancies including NSCLC and controls various cellular processes in different contexts, including tumor cell migration and invasion.^18^, ^19^ CCND2 is a well‐known cyclin that functions in the cell cycle, specifically in G1/S transition.^20^ Here, we verified that FGF2 and CCND2 were the direct targets of miR‐646 by dual‐luciferase assay, and the overexpression of miR‐646 significantly suppressed the expression of FGF2 and CCND2's mRNA and protein in NSCLC cell lines. Moreover, rescue assays demonstrated that FGF2 or CCND2 overexpressions could reverse the suppression of proliferation and invasion induced by miR‐646. Jointly, we established that the suppressive effects of miR‐646 on NSCLC are mediated by the downregulation of FGF2 and CCND2.

In conclusion, miR‐646 is downregulated in NSCLC and could suppress the proliferation and EMT‐induced metastasis of NSCLC by suppressing FGF2 and CCND2. Thus, downregulation of miR‐646 can play a central role in tumor progression and may be a potential therapeutic target for NSCLC.

## CONFLICT OF INTEREST

All the authors declare no conflict of interest, financial or otherwise.

## AUTHOR CONTRIBUTIONS

JW and SG conceived and designed the study, and also wrote and reviewed the manuscript. JW, HS, and SG acquired, analyzed, and interpreted the data. All the authors read and approved the final manuscript.

## Data Availability

The analyzed datasets generated during the study are available from the corresponding author on reasonable request.
